# Evaluation of radiomic feature harmonization techniques for benign and malignant pulmonary nodules

**Published:** 2025-01-15

**Authors:** Claire Huchthausen, Menglin Shi, Gabriel L.A. de Sousa, Jonathan Colen, Emery Shelley, James Larner, Einsley Janowski, Krishni Wijesooriya

**Affiliations:** 1 Department of Physics, University of Virginia; 2 Department of Biomedical Engineering, Northwestern University; 3 Joint Institute on Advanced Computing for Environmental Sciences, Old Dominion University; 4 Hampton Roads Biomedical Research Consortium; 5 Department of Radiation Oncology, University of Virginia

## Abstract

**Background:**

Conventional methods for detecting lung cancer early are often qualitative and subject to interpretation. Radiomics provides quantitative characteristics of pulmonary nodules (PNs) in medical images, but variability in medical image acquisition is an obstacle to consistent clinical application of these quantitative features. Correcting radiomic features’ dependency on acquisition parameters is problematic when combining data from benign and malignant PNs, as is necessary when the goal is to diagnose lung cancer, because acquisition effects may differ between them due to their biological differences.

**Purpose:**

We evaluated whether we must account for biological differences between benign and malignant PNs when correcting the dependency of radiomic features on acquisition parameters, and we compared methods of doing this using ComBat harmonization.

**Methods:**

This study used a dataset of 567 clinical chest CT scans containing both malignant and benign PNs. Scans were grouped as benign, malignant, or lung cancer screening (mixed benign and malignant). Preprocessing and feature extraction from ROIs were performed using PyRadiomics. Optimized Permutation Nested ComBat harmonization was performed on extracted features to account for variability in four imaging protocols: contrast enhancement, scanner manufacturer, acquisition voltage, focal spot size. Three methods were compared: harmonizing all data collectively in the standard manner, harmonizing all data with a covariate to preserve distinctions between subgroups, and harmonizing subgroups separately. A significant (*p* ≤ 0.05) Kruskal-Wallis test determined whether harmonization removed a feature’s dependency on an acquisition parameter. A LASSO-SVM pipeline was trained using acquisition-independent radiomic features to predict whether PNs were malignant or benign. To evaluate the predictive information made available by each harmonization method, the trained harmonization estimators and predictive model were applied to a corresponding unseen test set. Harmonization and predictive performance metrics were assessed over 10 trials of 5-fold cross validation.

**Results:**

Kruskal-Wallis defined an average 2.1% of features (95% CI: 1.9–2.4%) as acquisition-independent when data were harmonized collectively, 27.3% of features (95% CI: 25.7–28.9%) as acquisition-independent when harmonized with a covariate, and 90.9% of features (95% CI: 90.4–91.5%) as acquisition-independent when harmonized separately. LASSO-SVM models trained on data harmonized separately or with a covariate had higher ROC-AUC for lung cancer screening scans than models trained on data harmonized without distinction between benign and malignant tissues (Delong test, Holm-Bonferroni adjusted *p* ≤ 0.05). There was not a conclusive difference in ROC-AUC between models trained on data harmonized separately and models trained on data harmonized with a covariate.

**Conclusions:**

Radiomic features of benign and malignant PNs require different corrective transformations to recover acquisition-independent distributions. This can be done using separate harmonization or harmonization with a covariate. Separate harmonization enabled the greatest number of predictive features to be used in a machine learning model to retrospectively detect lung cancer. Features harmonized separately and features harmonized with a covariate enabled predictive models to achieve similar performance on lung cancer screening scans.

## Introduction

Lung cancer causes one in five cancer deaths worldwide.^1^ Detecting the cancer early has major implications. Patients whose lung cancer is detected at an early stage when the cancer is localized exhibit a 61% five-year relative survival, whereas those whose cancer is undetected until it has distant metastasis have a much lower relative survival rate of 7%.^2^ Even before metastasis, larger tumors lead to higher treatment-related toxicity.^3^ Standard practice for detecting lung cancer before symptoms develop is through screening with low-dose computed tomography (CT) scans, which produce high-detail three-dimensional images of the lung. Lung cancer screening (LCS) is typically performed annually for adults at risk for lung cancer. Physicians examine a LCS CT scan for abnormal growths or pulmonary nodules (PNs), which are marked as suspicious if they meet certain criteria.^4^ But common indicators such as size and rate of growth are sometimes caused by benign inflammatory processes,^5^ and other visual cues such as smoothness, irregularity and speculation are not quantitative but subject to each physician’s interpretation.^6^ Follow-up scans over a course of months and biopsy or Positron Emission Tomography (PET) scans are often required for a definitive diagnosis.

Medical images contain information indiscernible to the naked eye^7^ which could be used in quantitative diagnostic tools. The field of radiomics aims to extract such information into radiomic features, a set of complex statistical and spatial interrelationships between selected voxels in an image.^8^ These radiomic features can be correlated with clinical outcomes^9^ or serve as predictive biomarkers.^8^ Efforts to standardize the field and make radiomics a more reliable tool include PyRadiomics, an open-source Python library for medical image processing and extraction of radiomic features.^9^ Radiomic features remain sensitive to different scanners and other imaging protocols,^10^ which vary across and even within clinics. These non-biological sources of variation make it difficult to meaningfully combine data or reproduce results.^11^ This is a primary obstacle to using radiomics to predict cancer in PNs in a clinical context.

Methods to reduce effects of acquisition variability include ComBat (“Combine Batches”) harmonization. The ComBat algorithm is applied after data extraction to compensate for systematic biases in data, and has been shown to mitigate the effects of variable image acquisition parameters.^12–14^ Optimized Permutation Nested ComBat (OPNCB) extends this approach to remove biases from multiple imaging protocols sequentially, which is needed for large heterogeneous medical image datasets.^15^ However, applying harmonization becomes problematic when biological differences between PNs cause the corresponding images to be affected differently by variation in acquisition. To make predictive models suitable for a clinical context, we must remove the acquisition differences in a manner consistent with the biological differences between benign and malignant tissues.

ComBat can be applied with a covariate to account for the differences between subgroups of a dataset, such as benign vs. malignant scans.^16^ However, it has been demonstrated that different tissue types (breast tumor vs. healthy liver tissue) require distinct ComBat transformations.^17^ As a data-driven method, ComBat is specific to a tissue type, so different tissue types should be harmonized separately.^16^ We have asked here, if different tissue types must be harmonized separately, how must benign and malignant forms of the same tissue (lung tumor vs. benign lung tissue) be treated in harmonization? To our knowledge, this question has not yet been studied.

In CT-based radiomics for lung cancer, ComBat harmonization has been applied mostly in predictive pipelines addressing outcomes like recurrence,^18^ progression-free survival,^19^ and overall survival.^20^ These predictive problems use data from only malignant PNs. In contrast, lung cancer diagnosis pipelines require data from both benign and malignant PNs. One study which did apply ComBat harmonization to a dataset containing both benign and malignant PNs for lung cancer diagnosis did not make a distinction between benign and malignant PNs during harmonization.^21^ The study assumed a common bias to different data cohorts they used, but did not harmonize over variations in imaging protocols within each cohort.^21^

In this study, we have examined methods for harmonizing radiomic data from benign and malignant PNs to correct for dependency on acquisition parameters. We used radiomic features extracted using PyRadiomics from chest CT scans acquired from the hospital at our institution. To remove radiomic features’ dependency on four acquisition parameters, we compared three methods of applying nested ComBat: (i) harmonizing subgroups collectively in the standard manner, with no distinction between benign and malignant tissues, (ii) harmonizing with a covariate to preserve subgroups, and (iii) harmonizing subgroups separately. To compare the predictive information contained in successfully harmonized, acquisition-independent features, we used a feature selection and machine learning pipeline to predict lung cancer in PNs.

## Methods

### A. Image Acquisition and Segmentation

Chest CT images containing both benign and malignant PNs were acquired from 193 patients at our institution. In all, 305 unique PNs were included, with some patients having multiple PNs. A total of 567 three-dimensional chest CT images were acquired, as multiple scans were sometimes performed for these PNs over time. The contents of our dataset are summarized in Table 1. Physician annotations and diagnostic information such as biopsy and PET/CT results or written diagnosis from visual evidence were available for all PNs.

**Figure 1.**
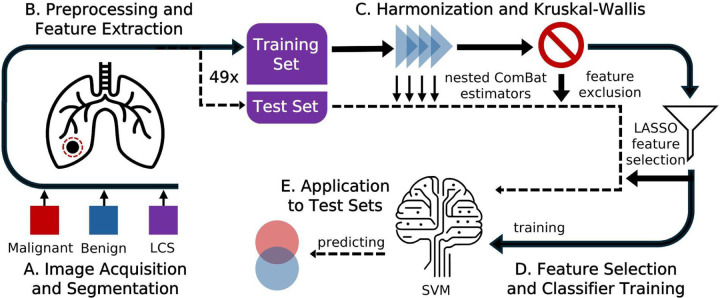
The radiomic workflow used in this study. The solid line represents the training set, and the dashed line represents the test set for a given trial.

We partitioned our data into 3 subgroups: a benign subgroup, a malignant subgroup, and a LCS subgroup (diagnosis at time of scan unknown). We partitioned scans known to be benign from scans known to be malignant in order to study how this distinction in PN biology affects harmonization. The benign group holds scans at all timepoints of PNs confirmed to be benign by biopsy or wedge resection. Some scans in this subgroup were noted by physicians as suspicious for malignancy prior to the diagnostic procedure. PNs whose scans are in the malignant subgroup had a visual diagnosis at the time of scan and were later confirmed to be malignant through biopsy or PET/CT. The LCS subgroup holds scans of PNs which were visually diagnosed by physicians as benign according to Lung-RADS qualifications.^4^ Some PNs in the LCS subgroup later developed malignancy. In these cases, later scans of the same PN after diagnosis are in the malignant subgroup. The other LCS PNs, with no future evidence of the PN becoming malignant, were presumed to be benign. A flowchart describing how a given scan was sorted into a subgroup is given in Figure S1. The breakdown of our dataset by subgroup is detailed in Table 1 and is noted in Figure 1A.

Prior to a CT scan, iodine-based contrast medium is sometimes administered to highlight the blood and other structures in the image.^22^ Contrast enhancement (CE) is a major factor to consider in radiomic data because it increases pixel gray level values, the basis of most radiomic feature calculations, to an extent detectable by the naked eye. Additionally, the accumulation of contrast agent in tumors is expected to be higher than that in healthy tissue.^23^ However, physicians are unlikely to prescribe CE for a screening scan; contrast is typically administered only when there is already cause for concern.^24^ This produces a quasi-systematic difference between malignant and benign data. A harmonization method must ensure that a retrospective diagnostic model does not merely detect downstream effects of CE. To allow harmonization over CE, we gathered a mixture of CE and non-CE data for each subgroup (Table S1).

All images were acquired in DICOM format. Contrast enhancement information was noted from physician comments and all other acquisition parameter information was extracted from the DICOM header files. Following physician annotations, each region of interest (ROI) corresponding to a PN in the CT images was segmented using semi-automated contouring tools in Varian's Velocity AI software. Pixel data and segmentations were extracted from the DICOM files.

### B. Preprocessing and Feature Extraction

Preprocessing and feature extraction in our radiomic workflow are indicated by Figure 1B. Radiomic texture features require voxels to be rotationally invariant.^8^ Using the PyRadiomics feature extractor,^9^ images and masks were resampled to 1.0 cubic mm voxels. Lanczos interpolation was used for the images for greatest reproducibility,^25^ and nearest-neighbor interpolation was used for segmentation masks. To ensure exclusion of air and bone voxels and improve reproducibility,^26^ voxels with intensities outside a threshold of −700 to 500 Hounsfield Units (HU) were excluded from calculations of non-shape features. Other radiomic studies of PNs^27–29^ have used narrower thresholds to analyze only malignant tissue,^28^ but we chose a less conservative threshold because we also analyzed benign tissue and very small PNs.

All other PyRadiomics feature extractor settings were left at default values. In total, 107 radiomic features were extracted from all scans.

### C. Harmonization and Kruskal-Wallis Test

The ComBat algorithm removes the bias of an imaging protocol from a sample feature value by learning corrective transformations from a pool of data. It assumes an acquisition parameter affects different radiomic features and different samples in similar ways.^11^ Because ComBat is a data-driven method, no phantom imaging is required for this standardization.^30^ A covariate is a corrective parameter learned for a specific subgroup. Using a covariate assumes that a given instance of an acquisition parameter has the same effect on scans in different subgroups; a transformation applied to members of different subgroups would have the same slope but different intercept.^16^ A covariate accounts for differing frequencies at which scans in different subgroups are acquired using that protocol,^16^ e.g., malignant scans are more likely than benign scans to be acquired using CE (Table S1). Orlhac et al. recommends using a covariate when these assumptions can be made, as this allows ComBat to draw on a larger sample size. On the other hand, when an imaging protocol affects different subgroups (e.g. tissue type) differently, those subgroups should be harmonized separately.^16^

We aimed to remove differences due to image acquisition in a manner accounting for the effects of biological differences between our defined subgroups. To this end, we compared the performance of harmonizing subgroups collectively with no distinction (hereafter: collective harmonization), harmonizing with a covariate to preserve subgroups (hereafter: covariate harmonization), or dividing the dataset into subgroups and harmonizing these subgroups separately (hereafter: separate harmonization).

OPNCB applies the ComBat algorithm iteratively, correcting for one acquisition parameter after another. Acquisition parameters for which we used harmonization to correct were kilovoltage peak (KVP), focal spot size, scanner manufacturer, and contrast enhancement (CE). The impacts of varying KVP,^10^ focal spots,^31^ manufacturers,^29,32^ and CE^33^ have been studied. All instances of these acquisition parameters used to acquire scans in our dataset are detailed in Table S1. The OPNCB algorithm was used to implement each harmonization method. In OPNCB, harmonization is performed for each acquisition parameter sequentially in an order which removes acquisition dependency from the most features, as compared to other permutations.^15^ The application of nested ComBat harmonization in our radiomic workflow is indicated in Figure 1C.

Radiomic data from all PNs were split into five folds using Stratified K-fold. The process was repeated 10 times, resulting in 50 unique training sets and corresponding test sets. The training and test sets in a given trial were identical for all harmonization methods. However, ComBat harmonization fails if too few samples were acquired with the same instance of an acquisition parameter, e.g., if only two scans in a subgroup were acquired using a certain focal spot size. In such cases, these scans were excluded from the dataset. If an instance of an acquisition parameter was used to acquire a scan in a test set which was not used to acquire any scan in the corresponding training set, these scans were also excluded from the dataset. A total of 10 scans were excluded entirely and are not included in Table 1. One trial was excluded because, due to random splitting, its training and test sets contained several scans with acquisition parameters which were too rare to harmonize in that trial. Thus 49 trials were included in total.

To test whether harmonization was successful, we applied the Kruskal-Wallis test to compare the distributions of harmonized features as grouped by a specific instance of an acquisition parameter.^30^ For example, when considering CE, we used Kruskal-Wallis to compare feature distributions from all enhanced scans to those from all non-enhanced scans. The test was performed for all acquisition parameters. A significant result (*p* ≤ 0.05) means samples are likely not drawn from the same distribution. Features from known benign PNs should come from the same distribution regardless of acquisition, and similarly for known malignant PNs. However, the radiomic feature distributions may differ between benign and malignant PNs. We thus performed Kruskal-Wallis separately on the malignant and benign subgroups respectively. If the test returned a significant result for a feature, for either subgroup, that feature was still dependent on that acquisition parameter, and thus was not harmonized successfully. We excluded these acquisition-dependent features from all further analysis in that trial. We performed Kruskal-Wallis on the unharmonized and the collective, covariate, and separate harmonization versions of each training set. The application of the Kruskal-Wallis test and resulting feature exclusion in our radiomic workflow is indicated in Figure 1C.

### D. Feature Selection and Classifier Training

The primary objective of radiomic feature harmonization is to produce datasets independent of acquisition variability, but as a secondary test, we also evaluated whether successfully harmonized radiomic features were predictive of malignancy. To this end, we implemented a predictive pipeline whose target was PN malignancy. The key performance measure was prediction quality on the LCS subgroup, where PN diagnosis at the time of the scan is unknown.

We created a predictive pipeline using the training set of each trial. To increase the generalization of a predictive model^34^ and better model clinical applicability, we used a least absolute shrinkage and selection operator (LASSO) to select a subset of predictive features from acquisition-independent features before training a model. The LASSO was applied to each harmonization version of a training set.

LASSO-selected features from the training set were used to train a linear Support Vector Machine (SVM).^35^ The same pipeline, with regularization parameters of α = 0.05 for LASSO and C = 1 for SVM, was repeated for all 49 training sets. These steps in our radiomic workflow are indicated by Figure 1D.

### E. Application to Test Sets

We applied the trained ComBat estimators, feature selection results, and linear SVM to the unseen test set for each trial, as indicated in Figure 1E. The ComBat estimators appropriate to the acquisition parameters of a given test sample were applied in the same order determined for the training set by the OPNCB algorithm. For separate harmonization, the estimates performed for a subgroup of the training set were applied to scans in the same subgroup in the corresponding test set.

## Results

**Figure 2.**
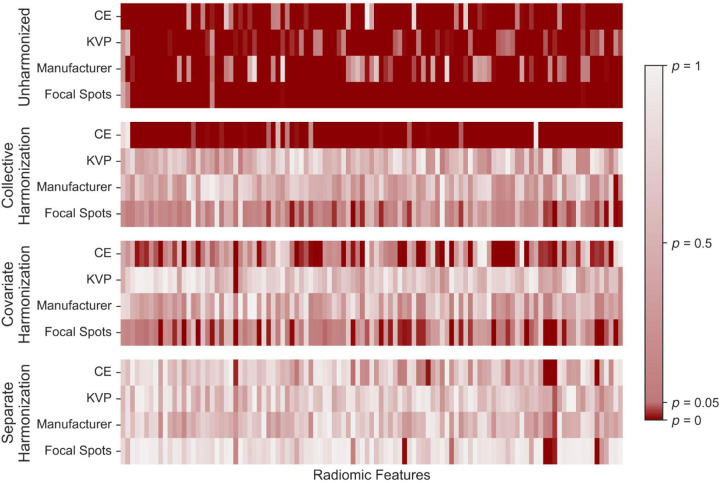
The *p*-values from the Kruskal Wallis test performed on an example training set for the malignant subgroup of each harmonization version respectively of the data. A significant *p*-value (≤ 0.05), indicating the remaining dependency of a feature (x-axes) on a given acquisition parameter (y-axes), is shown in dark red.

When comparing metrics for the three harmonization methods, adjusted *p*-values given below were calculated using Holm-Bonferroni. For unharmonized data, an average of 0.09% features (95% CI: 0.04–0.15%) showed no acquisition dependence according to the Kruskal-Wallis test. For standard collective harmonization, an average of 2.1% of features (95% CI: 1.9–2.4%) were acquisition-independent. For covariate harmonization, an average of 27.3% of features (95% CI: 25.7–28.9%) were acquisition-independent. For separate harmonization, an average of 90.9% of features (95% CI: 90.4–91.5%) were acquisition-independent. Separate harmonization produced statistically significant improvements compared to collective harmonization (*t-*test, adjusted *p* = 7.7e-88) and covariate harmonization (*t-*test, adjusted *p* = 8.1e-147). An example of the effects of harmonization on the malignant subgroup for a trial is shown in Figure 2 while the effects of harmonization on the benign and LCS subgroups are shown in Figures S2–S3. Out of the four acquisition parameters, CE was the most frequent source of acquisition dependency which remained after harmonization, for all harmonization methods, shown in Table 2. The frequency of acquisition dependence for each radiomic feature is reported in Table S2.

From the acquisition-independent features, LASSO selected an average of 1.1 features per trial (95% CI: 1.0–1.2) from collective harmonization features, 3.8 features per trial (95% CI: 3.5–4.1) from covariate harmonization features, and 7.4 features per trial (95% CI: 7.0–7.7) from separate harmonization features. Complete feature selection frequencies are given in Table S3. Elongation and Flatness were most frequently selected from acquisition-independent collective harmonization data, NGTDM Strength and Informational Measure of Correlation 1 were most frequently selected from acquisition-independent covariate harmonization data, and Sphericity and GLCM Inverse Difference were most frequently selected from acquisition-independent separate harmonization data. We show decision surfaces using these features from a sample trial in Figure S4. Some of these features have been used in radiomic models related to lung cancer,^36,37^ but further interpretation of these features is beyond the scope of this study.

**Figure 3.**
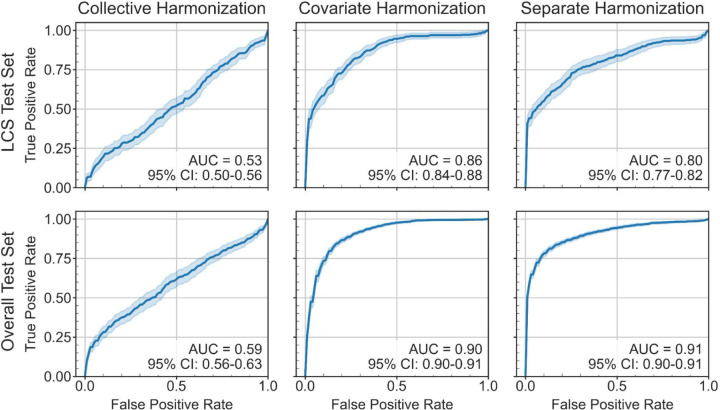
Mean receiver operating curves representing the testing performance of SVM models trained respectively on features selected by a LASSO (α = 0.05) from the corresponding training sets of each version of harmonized data. Each curve is the average over all trials with LASSO-selected acquisition-independent features (collective harmonization: 38 trials, covariate and separate harmonization: 49 trials) and the shaded regions show the 95% confidence interval. (Upper row) Mean receiver operating curves for only lung cancer screening (LCS) test samples. (Lower row) Mean receiver operating curves for all test samples.

LASSO-SVM models were trained on features harmonized using separate, covariate, or collective harmonization. We refer to these versions of the LASSO-SVM models respectively as separate, covariate, and collective harmonization models. The accuracy, sensitivity, and specificity of predictions for all test samples and for LCS test samples specifically are reported in Table 3 for all harmonization models. The corresponding ROC-AUCs for testing performance are shown in Figure 3. We focus on predictive performance for test samples in the LCS subgroup because these have unknown diagnosis at the time of scan, matching a clinical diagnosis scenario. For this subgroup, separate harmonization models outperformed covariate harmonization models in weighted accuracy (*t-*test, adjusted *p* = 4.8e-03) and specificity (*t-*test, adjusted *p* = 1.9e-11). Covariate harmonization models outperformed separate harmonization models in sensitivity (*t*-test, adjusted *p =* 1.4e-05). Collective harmonization models could not separate benign and malignant classes and classified all samples as benign. We compared the ROC-AUC of the models using the Delong test. The difference between separate and collective harmonization models was significant (adjusted *p* ≤ 0.05) for 26% of trials, in favor of separate harmonization. The difference between covariate and collective harmonization models was significant for 37% of trials, in favor of covariate harmonization. However, comparison of separate and covariate harmonization was inconclusive. Although the difference between separate and covariate harmonization models was significant for 45% of trials, covariate harmonization models had superior performance in 64% of significant trials, and separate harmonization models had superior performance in 36% of significant trials. Not reflected in these metrics is the fact that standard collective harmonization failed for 22% of trials to produce features which were acquisition-independent and predictive enough to be selected by a LASSO with α = 0.05 to train a model. Separate harmonization and covariate harmonization data successfully trained models in every trial.

## Discussion

In this work, we evaluated harmonization techniques for radiomic features extracted from benign and malignant PNs imaged with a range of acquisition protocols. The value of a radiomics-based diagnostic model is determined by its applicability to clinical scenarios, which often do not have standardized image acquisition. ComBat harmonization in radiomics aims to expand the usefulness of radiomic features in a clinical context by estimating the true distribution of the data independent of acquisition parameters. This is made problematic when trying to estimate the true distribution of two different kinds of data, such as benign vs. malignant PNs. Before a radiomic feature-based model can be built to diagnose lung cancer, we need to understand how harmonization must be applied when we have both benign and malignant data.

To our knowledge, it has not yet been studied how, or by how much, acquisition effects on radiomic features differ between benign and malignant PNs. For example, CE may have a large impact on features from malignant PNs, which have enhanced permeability and retention, while having a small effect on features from benign PNs. Because of such biological differences, we do not know if we can recover an acquisition-independent distribution by applying the same corrective transformations to radiomic features from both benign and malignant PNs. We aimed to understand whether scans of benign vs. malignant tissues need to be harmonized differently, in the same way that e.g. breast tissue vs. liver tissue need to be harmonized differently.^17^ To answer this question, we partitioned benign from malignant scans and evaluated methods of harmonizing them. Standard collective harmonization used the same corrective transformations for both benign and malignant subgroups. Separate and covariate harmonization allowed the ComBat algorithm to apply corrective transformations which are more specific to each subgroup. Separate harmonization gives the algorithm more freedom than covariate harmonization. We found that both separate and covariate harmonization recovered acquisition-independent distributions for significantly more features than collective harmonization, and separate harmonization recovered acquisition-independent distributions for three times as many radiomic features than covariate harmonization. These results indicate that radiomic features of benign and malignant PNs may be affected differently by acquisition parameters and need different corrective transformations to recover an acquisition-independent distribution.

Within our dataset, some scans shared the same sets of acquisition parameters, i.e., CE, scanner manufacturer, focal spots, and KVP were all uniform for some groups of scans. To gauge whether Kruskal-Wallis is useful to test for acquisition dependence, we applied the test to unharmonized data from these uniform groups. Our dataset contained 12 such sets of acquisition parameters, and these uniform groups ranged in size from 8 scans to 194 scans. Both benign and malignant PNs were represented in the group. We randomly split the unharmonized data from each uniform group into 5 folds for cross-validation, then considering benign and malignant PNs separately, we performed Kruskal-Wallis comparing each uniform training set and corresponding uniform test set. For scans taken with uniform acquisition parameters we do not expect the Kruskal-Wallis test to return a significant p-value. Indeed, Kruskal-Wallis performed on unharmonized data with uniform acquisition did not return a significant p-value for 94.0% of features (95% CI: 91.6–96.4%) when averaged over the 5 folds of cross-validation and over the 12 uniform groups. Thus Kruskal-Wallis appears to be a reasonable test to examine acquisition dependence of radiomic features. The results of the Kruskal Wallis test for separate harmonization data approached these results of the Kruskal Wallis test for unharmonized features with uniform acquisition parameters.

Our dataset was gathered from a clinical setting, and its high acquisition variability provided a realistic test of the examined harmonization methods. All three harmonization techniques were most challenged by CE (Table 2), which introduced a systematic bias between benign and malignant PNs, unlike the other three acquisition parameters which were examined. We note that our dataset is heterogeneous in several additional acquisition parameters (e.g. scanner model and convolution kernel^32^) which could not be controlled for in this study. Our dataset was not large enough to support harmonization over these highly variable parameters. Despite this limitation, none of these parameters systematically biased the data, as CE did. As noted above, we found that the Kruskal-Wallis test on scans with uniform CE, manufacturer, focal spots, and KVP still returned a significant *p-*value for a (small) nonzero number of features, and variability in unaccounted-for acquisition parameters most likely caused this deviation from a normal distribution. Type of lung cancer (e.g. squamous cell, adenocarcinoma) for malignant scans was also not uniform and was another source of variability for malignant PNs. Finally, because these scans were acquired as part of true diagnostic and screening processes, some images have unknown diagnoses at the time of scan. We accounted for this by partitioning such scans into our LCS subgroup. For LCS scans which we labeled benign in our predictive models, we had no confirmation they were benign except the absence of a later cancer diagnosis. For LCS scans which we labeled malignant in our predictive models, though they were later diagnosed as malignant, we could not know whether these were already malignant at scan time. This was a limitation in using predictive performance on this group to evaluate predictive information. However, scans in the LCS group are what is actually seen in a clinical diagnosis scenario and were appropriate for evaluating the predictive power of harmonized radiomic features.

Because we want an understanding of harmonization to enable future diagnostic models, an important factor to examine is whether features made acquisition-independent by these methods can predict malignancy. Recovering an acquisition-independent data distribution may not increase diagnostic performance. When comparing our separate harmonization models to ones trained on unharmonized data (Table S4), we found no significant difference for LCS accuracy (*t*-test, *p* = 0.22), LCS sensitivity (*t*-test, *p* = 0.33), and LCS specificity (*t*-test, *p* = 0.08). However, models trained on unharmonized data are not guaranteed to achieve similar accuracy at new acquisition sites.^11,16^ Their accuracy in this case may be an artifact of acquisition effects that differ distinctively between benign and malignant PNs, which effects are the targets of harmonization to remove. Thus, increasing predictive performance alone was not our goal in this study of harmonization. Indeed, there is no guarantee that all successfully harmonized radiomic features are predictive of malignancy. Collective harmonization yielded a small number of acquisition-independent features that could not be used to separate benign and malignant PNs. Instead, the collective harmonization models classified all test samples as benign (Table 3). We aimed to identify harmonization methods which can remove acquisition dependency from features which are predictive of malignancy.

The most rigorous test of predictive performance was a model’s performance on the LCS subgroup, with unknown diagnosis at time of scan. We found that covariate and separate harmonization models outperformed collective harmonization models on these scans. Separate harmonization successfully removes a subgroup’s dependency on acquisition parameters using corrective transformations completely unique to that subgroup. However, in doing so it may also introduce some artificial distinction between benign and malignant subgroups while accounting for the true biological distinction. This may make diagnosis more difficult for PNs which develop cancer in the future and do not fit cleanly into the malignant subgroup. This may explain separate harmonization models’ lower specificity for test samples in the LCS subgroup.

Separate harmonization and covariate harmonization both made predictive radiomic features acquisition-independent and allowed these features to be used in a predictive model. As a result, we recommend that radiomic features corresponding to benign vs. malignant PNs be harmonized either separately or with a covariate. Since one performed better in sensitivity and the other performed better in specificity, we cannot recommend one over the other based on our current analysis. Evaluation of these methods on another lung cancer dataset to form a more decisive conclusion are a direction of future study. Furthermore, our results may reflect fundamental differences between acquisition effects on radiomic features of benign vs. malignant tissues more generally. Evaluation of these methods for other cancer types is another direction of future study.

Before radiomics can be used to diagnose lung cancer in a real-world clinical setting, we must establish methods to remove acquisition dependency of radiomic features of both benign and malignant tissues. Investigating these harmonization methods is an important step towards being able to use radiomics as a quantitative tool for cancer diagnosis, which could allow malignant tumors to be treated early as well as reduce unnecessary resection and imaging procedures.

## Conclusion

We applied nested ComBat harmonization to CT scans of malignant and benign PNs in three manners. Harmonizing subgroups separately removed dependency on acquisition parameters for more features (90.9%) than harmonizing with a covariate (27.3% features) or harmonizing collectively (2.1% features). Separate and covariate harmonization both allowed predictive features to be used to predict lung cancer from screening scans with comparable average accuracy and ROC-AUC. We recommend that radiomic features of benign and malignant PNs be harmonized separately or with a covariate to remove acquisition dependency.

## Supplementary Material

Supplement 1

## Figures and Tables

**Table 1.** The contents of the dataset organized by subgroup. Patients and PNs in the LCS subgroup which later developed malignancy are also included in the malignant subgroup at later time points.

	Patients		Pulmonary Nodules		Scans	
	Count	% Malignant	Count	% Malignant	Count	% Malignant
LCS	102	15.7	224	7.6	323	9.9
Benign	27	0.0	37	0.0	58	0.0
Malignant	56	100.0	57	100.0	186	100.0
Overall	193	31.0	305	20.0	567	38.4

**Table 2.** The frequency by which harmonization failed to remove dependency of a feature on a given acquisition parameter, as determined by the Kruskal-Wallis test (*p ≤* 0.05) performed on the cancer and benign subgroups, for three harmonization methods. The given frequencies are the number of eliminations due to the respective acquisition parameters over all features (107) and all trials (49). Some features remained dependent on multiple acquisition parameters and/or were acquisition dependent for both subgroups.

	Separate Harmonization (%)		Covariate Harmonization (%)		Collective Harmonization (%)	
	Malignant	Benign	Malignant	Benign	Malignant	Benign
Focal Spots	6.1	2.3	41.9	20.4	27.8	11.7
KVP	0.6	0.5	3.2	5.8	3.2	7.6
Manufacturer	1.6	0.2	17.7	8.7	8.4	7.6
CE	7.2	0.8	38.9	38.3	92.3	66.7
Subgroup Total	8.9	2.8	55.6	53.3	93.8	77.0
Method Total	9.1		72.7		98.0	

**Table 3.** Average metrics for Support Vector Machines trained on LASSO-selected features from each harmonization version of the data. Weighted accuracies are reported with balanced weighting by class (benign/malignant). For collective harmonization, metrics are reported only for trials with LASSO-selected acquisition-independent features.

	Collective Harmonization (38 trials)		Covariate Harmonization (49 trials)		Separate Harmonization (49 trials)	
	Estimate, %	95% CI, %	Estimate, %	95% CI, %	Estimate, %	95% CI, %
LCS Weighted Accuracy	50.0	(50.0, 50.0)	75.6	(73.3, 77.9)	70.8	(68.5, 73.2)
LCS Sensitivity	0.0	(0.0, 0.0)	60.8	(56.3, 65.3)	45.6	(40.7, 50.5)
LCS Specificity	100.0	(100.0, 100.0)	90.3	(89.0, 91.6)	96.1	(95.3, 96.8)
Overall Weighted Accuracy	50.0	(50.0, 50.0)	82.9	(81. 9, 83. 9)	82.4	(81.6, 83.3)
Overall Sensitivity	0.0	(0.0, 0.0)	84.1	(82.2, 86.0)	71.0	(69.1, 72. 9)
Overall Specificity	100.0	(100.0, 100.0)	81.6	(79.9, 83.4)	93.8	(92.9, 94.7)
