# Supplementary material for: Evaluation of radiomic feature harmonization techniques for benign and malignant pulmonary nodules
Source: ArXiv. 2025 Jan 15:arXiv:2412.16758v2. Preprint. [Version 2] (PMC11774441)
Supplement: Supplement 1 [file NIHPP2412.16758v2-supplement-1.pdf]

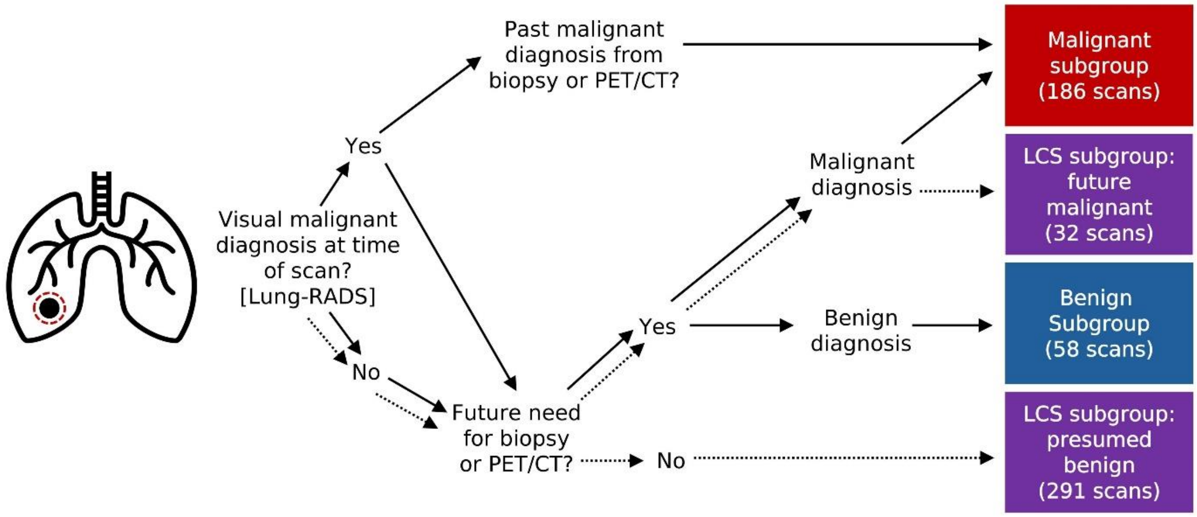

Figure S1. A flowchart describing how the dataset was partitioned into subgroups.

Supplemental Material

Table S1. The number of scans with each instance of all acquisition protocols, by subgroup.				
Protocol	Instance	Malignant	Benign	LCS
Contrast Enhancement	0	54	26	310
	1	132	32	13
Focal Spots	0.7	81	14	166
	0.8	10	12	32
	0.9	6	0	67
	1.2	89	32	49
	[1.6, 1.6]	0	0	9
Kilovoltage Peak	100	17	13	0
	110	0	0	9
	120	169	45	314
Manufacturer	GE Medical Systems	160	25	194
	SIEMENS	26	33	129

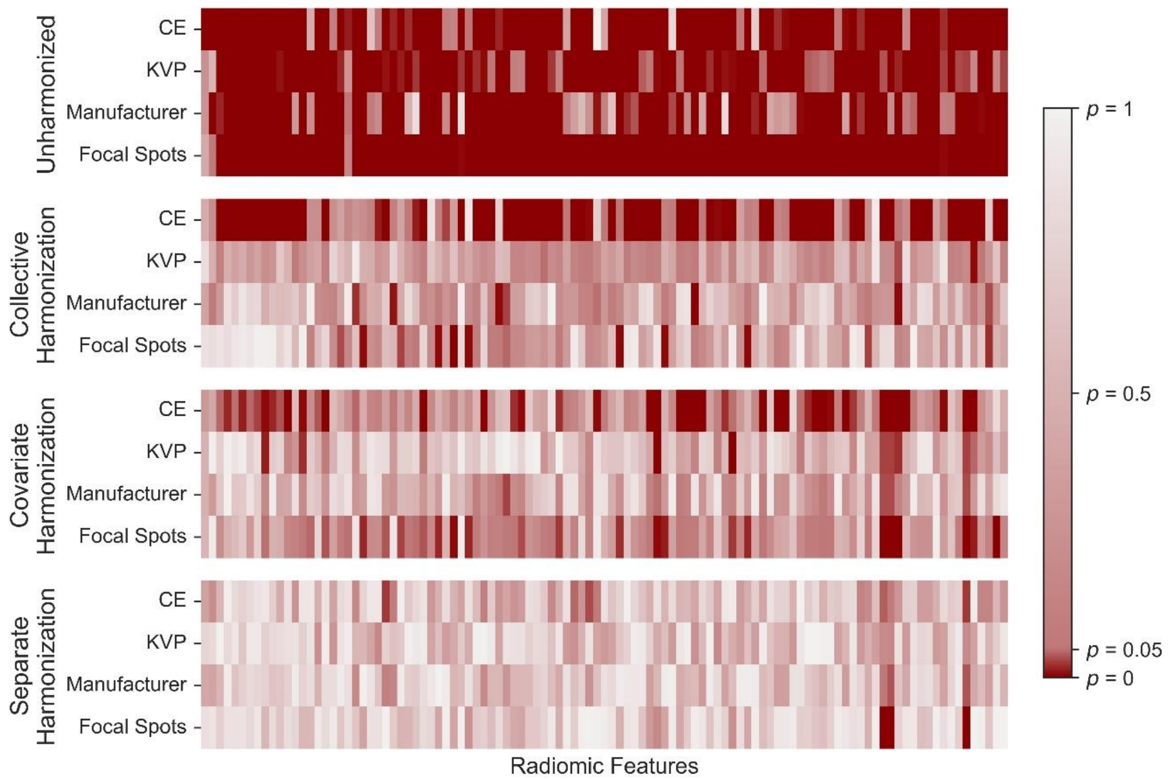

Figure S2. The *p*-values from the Kruskal Wallis test performed on an example training set for the benign subgroup of each harmonization version respectively of the data. A significant *p*-value (≤ 0.05), indicating the remaining dependency of a feature (x-axes) on a given acquisition parameter (y-axes), is shown in dark red.

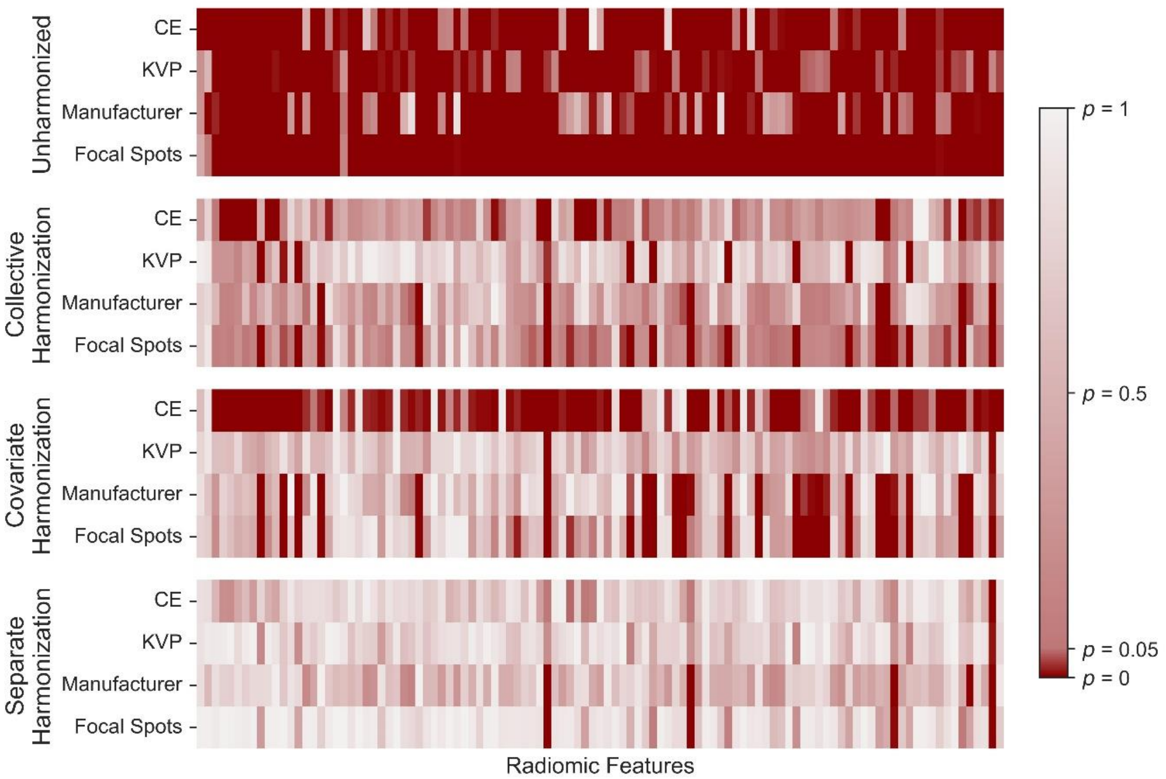

Figure S3. The *p*-values from the Kruskal Wallis test performed on an example training set for the LCS subgroup of each harmonization version respectively of the data. A significant *p*-value (≤ 0.05), indicating the remaining dependency of a feature (x-axes) on a given acquisition parameter (y-axes), is shown in dark red.

Table S2. Elimination frequency by the Kruskal-Wallis test ($p \leq 0.05$) performed on the malignant and benign subgroups for all radiomic features and three harmonization methods. The given frequencies are the number of eliminations over all trials (49).						
	Separate Harmonization (%)		Covariate Harmonization (%)		Collective Harmonization (%)	
	Malignant	Benign	Malignant	Benign	Malignant	Benign
shape Elongation			49.0		44.9	
shape Flatness			34.7		2.0	8.2
shape Least Axis Length			2.0	12.2	100.0	100.0
shape Major Axis Length			100.0	59.2	100.0	100.0
shape Maximum 2D Diameter Column			91.8	55.1	100.0	100.0
shape Maximum 2D Diameter Row			100.0	51.0	100.0	100.0
shape Maximum 2D Diameter Slice			30.6	30.6	100.0	100.0
shape Maximum 3D Diameter			100.0	44.9	100.0	100.0
shape Mesh Volume	10.2		98.0	81.6	100.0	100.0
shape Minor Axis Length			30.6	38.8	100.0	100.0
shape Sphericity			14.3	89.8	100.0	100.0
shape Surface Area			95.9	95.9	100.0	100.0
shape Surface Volume Ratio					100.0	100.0
shape Voxel Volume	6.1		98.0	79.6	100.0	100.0
first order 10 Percentile				36.7	100.0	10.2
first order 90 Percentile			12.2	51.0	57.1	28.6
first order Energy			91.8	89.8	100.0	100.0
first order Entropy			40.8	24.5	100.0	18.4
first order Interquartile Range			36.7	55.1	100.0	55.1
first order Kurtosis			95.9	53.1	100.0	6.1
first order Maximum			18.4	69.4	100.0	32.7
first order Mean Absolute Deviation			71.4	83.7	87.8	89.8
first order Mean			10.2	20.4	100.0	4.1
first order Median				20.4	100.0	51.0
first order Minimum	95.9	8.2	100.0	24.5	100.0	100.0
first order Range			10.2	67.3	100.0	59.2
first order Robust Mean Absolute Deviation			57.1	69.4	100.0	53.1
first order Root Mean Squared			8.2	18.4	100.0	24.5
first order Skewness				16.3	100.0	77.6
first order Total Energy			91.8	89.8	100.0	100.0
first order Uniformity			26.5	51.0	100.0	20.4
first order Variance			73.5	95.9	69.4	93.9
GLCM Autocorrelation				4.1	100.0	55.1
GLCM Cluster Prominence			18.4	100.0	8.2	98.0
GLCM Cluster Shade					100.0	98.0
GLCM Cluster Tendency			20.4	87.8	20.4	93.9
GLCM Contrast			100.0	61.2	100.0	100.0
GLCM Correlation			100.0	100.0	93.9	100.0
GLCM Difference Average			93.9	36.7	100.0	100.0
GLCM Difference Entropy			18.4	46.9	100.0	85.7
GLCM Difference Variance			95.9	93.9	30.6	100.0

GLCM Id			100.0	100.0	100.0	100.0
GLCM Idm			100.0	100.0	100.0	100.0
GLCM Idmn			63.3	2.0	100.0	100.0
GLCM Idn			73.5		100.0	100.0
GLCM Imc1			8.2		100.0	100.0
GLCM Imc2			16.3	2.0	100.0	98.0
GLCM Inverse Variance			100.0	100.0	100.0	100.0
GLCM Joint Average				36.7	100.0	20.4
GLCM Joint Energy	26.5	2.0	98.0	63.3	100.0	100.0
GLCM Joint Entropy			44.9		100.0	100.0
GLCM MCC		6.1	100.0		100.0	91.8
GLCM Maximum Probability		2.0	53.1	89.8	100.0	32.7
GLCM Sum Average				36.7	100.0	20.4
GLCM Sum Entropy			6.1	14.3	100.0	100.0
GLCM Sum Squares			91.8	89.8	100.0	95.9
GLDM Dependence Entropy			6.1		100.0	100.0
GLDM Dependence Non-Uniformity			89.8	4.1	100.0	100.0
GLDM Dependence Non-Uniformity Normalized			28.6	4.1	100.0	100.0
GLDM Dependence Variance	24.5		100.0	100.0	100.0	100.0
GLDM Gray Level Non-Uniformity	93.9		100.0	93.9	100.0	100.0
GLDM Gray Level Variance			73.5	95.9	71.4	93.9
GLDM High Gray Level Emphasis				16.3	100.0	4.1
GLDM Large Dependence Emphasis	42.9		100.0	100.0	100.0	100.0
GLDM Large Dependence High Gray Level Emphasis	10.2		100.0	100.0	100.0	100.0
GLDM Large Dependence Low Gray Level Emphasis	81.6		77.6	100.0	93.9	85.7
GLDM Low Gray Level Emphasis			2.0	98.0	100.0	46.9
GLDM Small Dependence Emphasis			75.5	6.1	100.0	100.0
GLDM Small Dependence High Gray Level Emphasis*	4.1		100.0	38.8	98.0	95.9
GLDM Small Dependence Low Gray Level Emphasis			2.0	67.3	100.0	95.9
GLRLM Gray Level Non Uniformity	81.6		100.0	89.8	100.0	100.0
GLRLM Gray Level Non-Uniformity Normalized			2.0	38.8	100.0	16.3
GLRLM Gray Level Variance			71.4	98.0	75.5	95.9
GLRLM High Gray Level Run Emphasis			10.2	22.4	100.0	4.1
GLRLM Long Run Emphasis	8.2		100.0	98.0	100.0	100.0
GLRLM Long Run High Gray Level Emphasis					100.0	100.0
GLRLM Long Run Low Gray Level Emphasis			2.0	100.0	100.0	22.4
GLRLM Low Gray Level Run Emphasis			2.0	98.0	100.0	34.7
GLRLM Run Entropy			8.2	8.2	100.0	100.0

GLRLM Run Length Non-Uniformity			100.0	34.7	100.0	100.0
GLRLM Run Length Non-Uniformity Normalized			100.0	71.4	100.0	100.0
GLRLM Run Percentage			100.0	93.9	100.0	100.0
GLRLM Run Variance	57.1		100.0	100.0	100.0	100.0
GLRLM Short Run Emphasis			100.0	81.6	100.0	100.0
GLRLM Short Run High Gray Level Emphasis			38.8	34.7	100.0	
GLRLM Short Run Low Gray Level Emphasis			2.0	98.0	100.0	40.8
GLSZM Gray Level Non Uniformity			100.0	32.7	100.0	100.0
GLSZM Gray Level Non-Uniformity Normalized	8.2		79.6	34.7	100.0	98.0
GLSZM Gray Level Variance			34.7	77.6	8.2	79.6
GLSZM High Gray Level Zone Emphasis			73.5	46.9	100.0	
GLSZM Large Area Emphasis	100.0	95.9	100.0	100.0	100.0	100.0
GLSZM Large Area High Gray Level Emphasis	100.0	91.8	100.0	100.0	100.0	100.0
GLSZM Large Area Low Gray Level Emphasis	100.0		100.0	100.0	71.4	100.0
GLSZM Low Gray Level Zone Emphasis			4.1	98.0	100.0	4.1
GLSZM Size Zone Non-Uniformity			95.9	8.2	100.0	100.0
GLSZM Size Zone Non-Uniformity Normalized			4.1	4.1	100.0	100.0
GLSZM Small Area Emphasis			4.1	12.2	100.0	100.0
GLSZM Small Area High Gray Level Emphasis			79.6	55.1	100.0	16.3
GLSZM Small Area Low Gray Level Emphasis			2.0	87.8	100.0	26.5
GLSZM Zone Entropy			12.2		100.0	100.0
GLSZM Zone Percentage			83.7	8.2	100.0	100.0
GLSZM Zone Variance	100.0	95.9	100.0	100.0	100.0	100.0
NGTDM Busyness			98.0	100.0	100.0	100.0
NGTDM Coarseness			77.6		100.0	100.0
NGTDM Complexity	6.1		100.0	16.3	100.0	61.2
NGTDM Contrast			100.0	93.9	100.0	100.0
NGTDM Strength			16.3		100.0	100.0

Table S3. Selection frequency for radiomic features for three harmonization methods. Selection was performed by a LASSO (α = 0.05) on acquisition-independent features for a given trial. The given frequencies are the number of selections over all trials (49). Features which were never selected are not displayed.

	Separate Harmonization (%)	Covariate Harmonization (%)	Collective Harmonization (%)
shape Elongation	4.1	28.6	26.5
shape Flatness	6.1	57.1	53.1
shape Major Axis Length	2.0		
shape Sphericity	100.0	10.2	
first order 90 Percentile		2.0	
GLCM Correlation	87.8		
GLCM Difference Variance	34.7		
GLCM Id	98.0		
GLCM Idm	6.1		
GLCM Imc1		91.8	
GLCM Imc2		69.4	
GLCM Inverse Variance	71.4		
GLDM Dependence Entropy		6.1	
GLDM Dependence Non-Uniformity Normalized		18.4	
GLDM Dependence Variance	69.4		
GLDM Gray Level Variance			2.0
GLDM Small Dependence Emphasis	38.8	10.2	
GLDM Small Dependence High Gray Level Emphasis	95.9		
GLSZM Gray Level Variance	2.0		6.1
GLSZM Small Area Emphasis	87.8	2.0	
GLSZM Small Area High Gray Level Emphasis	4.1		
NGTDM Strength	28.6	83.7	

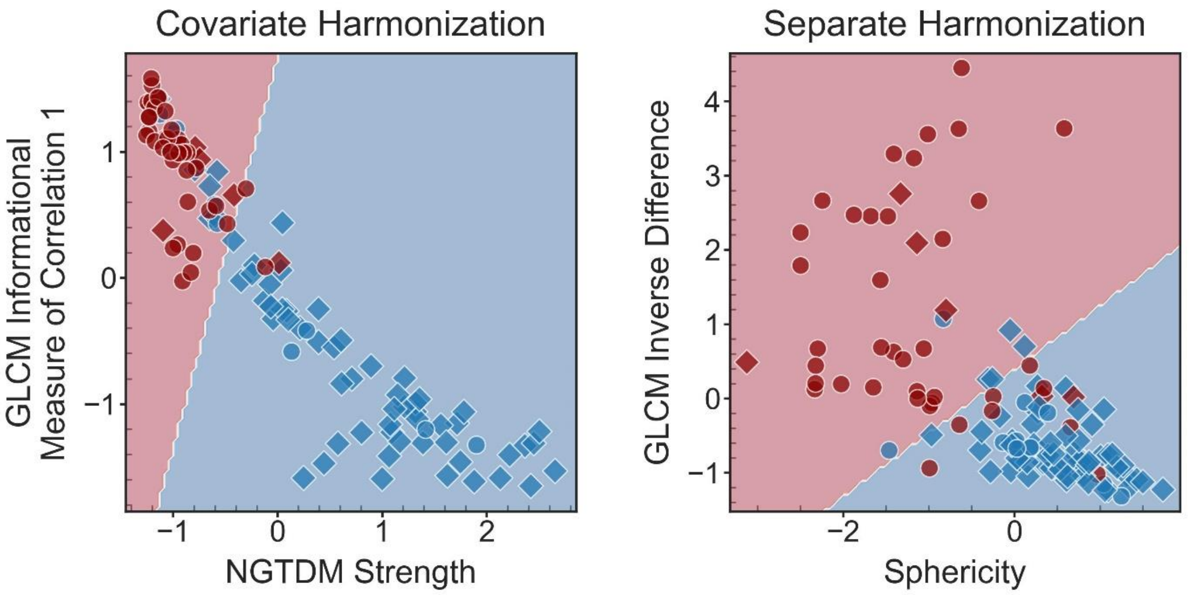

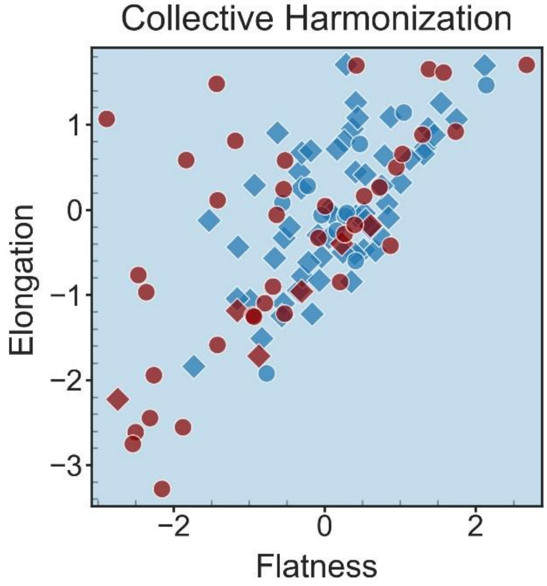

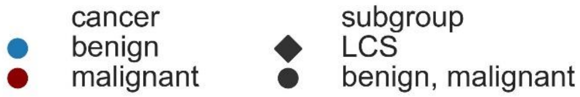

Figure S4. Example decision surfaces of an SVM model trained on two of the features selected by a LASSO (α = 0.05) for each harmonization version of the data. The radiomic features are scaled. Each test sample represents a pulmonary nodule. The SVM labels a test sample falling in a red area as malignant, and it labels a test sample falling in a blue area as benign. Marker colors denote the correct diagnosis for each test sample. We label LCS pulmonary nodules which develop malignancy in the future as malignant. (Left) The SVM trained on acquisition-independent collectively harmonized data could not separate benign and malignant classes.

Table S4. Metrics from LASSO-SVM trained on unharmonized data. Weighted accuracies are reported with balanced weighting by class (benign/malignant).		
	Estimate, %	95% CI, %
LCS Weighted Accuracy	71.9	(70.0, 74.2)
LCS Sensitivity	48.9	(44.2, 53.7)
LCS Specificity	94.9	(93. 9, 96.0)
Overall Weighted Accuracy	85.4	(84.6, 86.2)
Overall Sensitivity	80.2	(78.9, 81.6)
Overall Specificity	90.5	(89.4, 91.7)
